# Magnetic particle imaging tracers for human applications: preclinical in vivo evaluation of Magtrace, Resotran, and FerroTrace

**DOI:** 10.1038/s44303-026-00182-7

**Published:** 2026-07-21

**Authors:** Olivia C. Sehl, Nitara Fernando, Petrina Kim, A. Rahman Mohtasebzadeh, Toby Sanders, Kelvin Guo, Benjamin Fellows, Michael D. Alvarado, Benjamin Thierry, Marcela Weyhmiller, Joan M. Greve, Stephen Y. Lai, Patrick W. Goodwill, Paula J. Foster

**Affiliations:** 1 Magnetic Insight Inc, Alameda, CA USA; 2 Department of Medical Biophysics, Western University, Robarts Research Institute, London, ON Canada; 3 Department of Surgery, University of California San Francisco, San Francisco, CA USA; 4 Future Industries Institute, Adelaide University, Adelaide, Australia; 5 Department of Head and Neck Surgery, University of Texas MD Anderson Cancer Center, Houston, TX USA

**Keywords:** Cancer, Medical research, Oncology

## Abstract

Sentinel lymph node (SLN) mapping is essential for cancer staging and treatment planning. Superparamagnetic iron oxides (SPIOs) are used for magnetic SLN localization in breast cancer and are being explored in cancers with complex lymphatic drainage pathways. Magnetic particle imaging (MPI) directly detects SPIOs and has demonstrated feasibility for quantitative lymphography in mouse models. However, most MPI studies have relied on preclinical SPIO formulations not intended for human use. Here, we evaluated three clinically available or investigational SPIOs for MPI, including Magtrace, Resotran, and FerroTrace. SPIOs were characterized in vitro using 2D MPI and relaxometry, benchmarked against the preclinical standard VivoTrax. Magtrace and Resotran demonstrated in vitro performance comparable to VivoTrax, whereas FerroTrace exhibited lower peak signal and resolution by relaxometry. In vivo longitudinal MPI lymphography was performed in healthy C57BL/6 mice following intradermal injection of SPIOs. Magtrace and FerroTrace produced robust lymph node labeling, with MPI signal persisting for at least four weeks across all SPIOs. Feasibility of clinical-scale MPI for complex lymphatic mapping was demonstrated using a human-sized head-and-neck phantom modeling bilateral lymphatic drainage. Together, these results establish Magtrace, Resotran, and FerroTrace as suitable magnetic tracers for MPI lymphography and support their use in future human MPI applications.

## Introduction

Sentinel lymph node (SLN) mapping procedures are used to identify the first draining lymph node(s) from a solid tumor, which are most likely to harbor metastasis, to guide surgical and/or radiation treatment planning^1–8^. SLN mapping is an established standard of care in several cancers, including breast cancer and melanoma, and is increasingly being adopted in head and neck cancer^1–5^. Current workflows commonly employ peritumoral injection of radiotracers, which are taken up by lymphatic channels that drain into SLNs, then imaging with scintigraphy or SPECT/CT is performed to identify the nodal basins requiring intervention. For SLN biopsy (SLNB), a handheld gamma probe is used to identify nodes with the highest radioactive counts, which are then excised for staging by pathology. Nuclear medicine techniques are limited by the short radiotracer half-life (6 h for Tc-99m), which requires the radiotracer injection to be precisely timed with imaging and surgery, typically on the same day. They are also susceptible to the “shine-through” phenomenon, where high radioactivity at the injection site can obscure detection of adjacent SLNs, particularly in melanoma and head and neck cancers^7,9,10^.

The use of superparamagnetic iron oxides (SPIOs) has emerged as a promising non-radioactive alternative for use in SLNB and has been found to be non-inferior to radioisotope-guided SLNB in breast cancer^11–15^. In a magnetic SLNB procedure, magnetometer-based detectors are used to localize SPIO-labeled nodes intraoperatively. Magtrace® is a carboxydextran-coated SPIO approved across the world for SLN mapping in breast cancer^15,16^, with additional exploration in melanoma^17–20^ and oral cavity cancer^21–23^. FerroTrace® is a mannose-functionalized SPIO currently under clinical investigation in Australia for SLN mapping^24–26^ and has recently completed a phase I study in oral squamous cell carcinoma^27^, with additional trials underway in gastrointestinal cancers (NCT05038098, NCT05899985, ACTRN12621000748819). However, magnetometer-only approaches do not provide preoperative spatial imaging, which is essential for melanoma and head and neck cancers, where lymphatic drainage can be complex and involve multiple and/or unexpected nodal basins^4,28^.

Magnetic particle imaging (MPI) is an emerging imaging modality that directly detects SPIOs as “magnetic tracers” with zero tissue background^29^. By providing three-dimensional, quantitative, spatially resolved maps of magnetic tracer distribution, MPI has the potential to visualize draining SLNs and lymphatic pathways preoperatively^30,31^. Many of the SPIOs used as magnetic tracers for MPI are contrast agents used for magnetic resonance imaging (MRI)^32^, some of which have a history of more than 20 years of human use, including ferucarbotran, ferumoxide, ferumoxtran, and ferumoxytol^33^. Ferucarbotran remains approved for clinical MRI in Japan (Resovist®)^34,35^ and in Germany and Sweden (Resotran®)^36^, which have demonstrated similar MPI performance^37,38^. Ferumoxytol (Feraheme®) has Health Canada and U.S. FDA approval for the treatment of iron-deficiency anemia^39^ and has been used off-label as an MRI contrast agent^40–42^, prior to subsequent FDA approval of Ferabright® for visualizing brain lesions by MRI^43^. Ferumoxtran (Ferrotran®) is being tested in Europe in clinical trials to detect lymph node metastases with MRI^44,45^. However, ferumoxytol and ferumoxtran are ultrasmall SPIOs with cores that are too small to magnetize sufficiently for MPI, leading to low signal and resolution^37,46,47^.

As MPI advances toward human-scale scanners^48–50^ and clinical translation, there is a growing need to identify readily available magnetic tracers with established safety profiles and regulatory pathways for human applications. In this study, we compare the MPI performance and in vivo lymphatic pharmacokinetics of Magtrace, FerroTrace, and Resotran in murine models. Neither Magtrace nor FerroTrace have been previously evaluated for MPI. We compare these to VivoTrax, a preclinical SPIO with similar properties to Resovist, which has been widely used in preclinical MPI studies but is not intended for human use (Table 1). This builds on prior work showing that VivoTrax can be used to sensitively, specifically, and quantitatively identify and map SLNs in murine models in multiple regional lymphatic basins^30,31^. In addition, we use a human-sized head and neck phantom to illustrate how MPI could be applied for clinical SLN imaging.

**Table 1:** Reported physicochemical characteristics of VivoTrax, Magtrace, Resotran, and FerroTrace used in this study

	VivoTrax	Magtrace	Resotran	FerroTrace
Description	Superparamagnetic iron oxide dextran magnetite nanoparticles	Carboxydextran-coated superparamagnetic iron oxide particles	Ferucarbotran (Superparamagnetic iron oxide nanoparticles coated with carboxydextran)	Superparamagnetic iron oxide nanoparticles coated with block-copolymers
Formulation	Saline	Water for injection containing 0.3% w/v sodium chloride	Water for injection containing S-Lactic acid, mannitol, sodium hydroxide	Isotonic saline containing 0.9% w/v sodium chloride
Core size	4.2 nm	3.5–10 nm	5.8 ± 2.5 nm	16 ± 4 nm
Hydrodynamic diameter	62 nm (mean)	45–65 nm	57.4 ± 2.1 nm	73.9 nm
Reference(s)	^86^	^15^	^36,38^	^24,51^

Manufacturer-reported specifications are listed for each SPIO, including description, formulation, core size, and hydrodynamic diameter.

## Results

### Characterization of Magtrace, Resotran, and FerroTrace for MPI

**Fig. 1:**
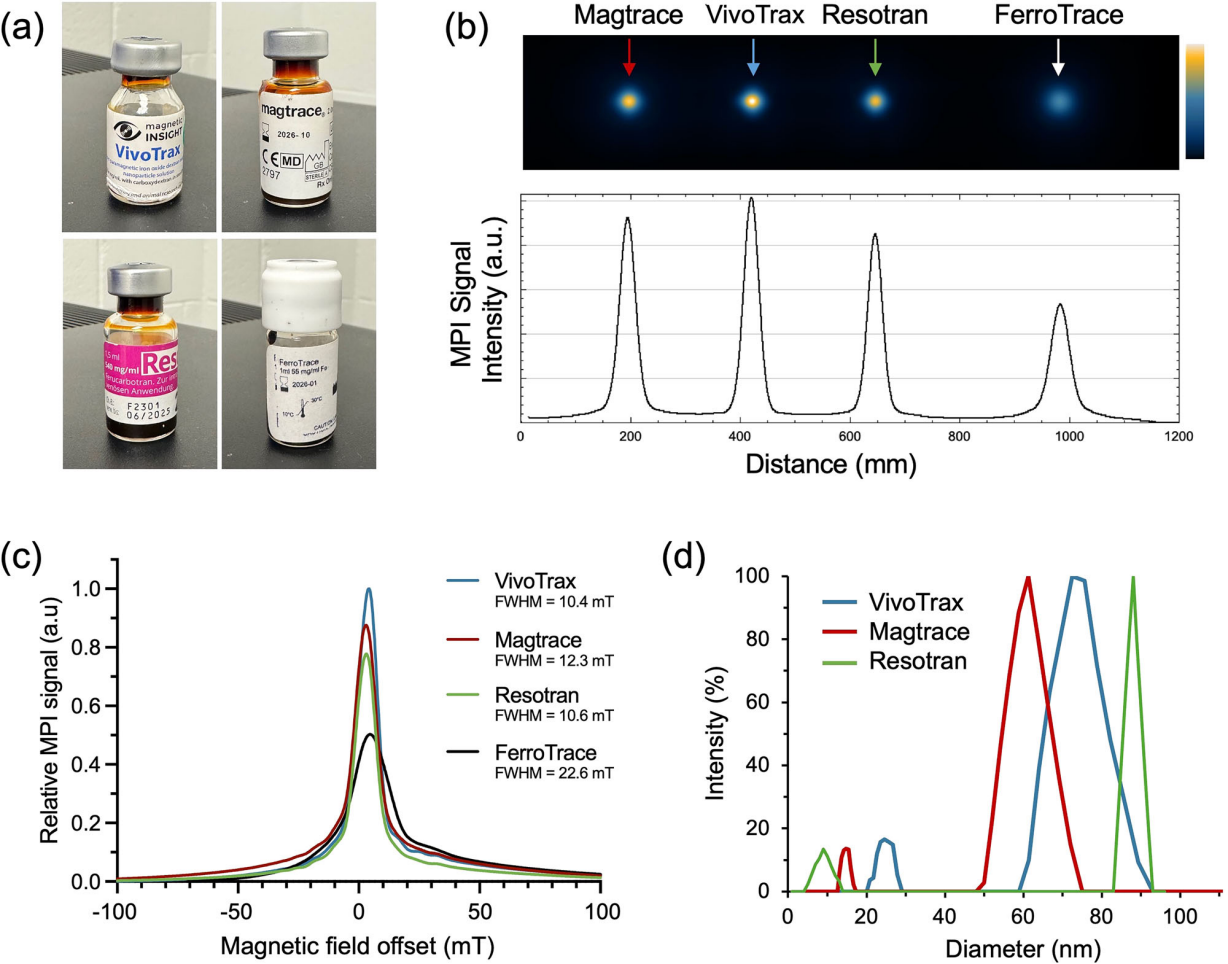
Characterization of Magtrace, Resotran, and FerroTrace benchmarked against VivoTrax. **a** Photo of VivoTrax, Magtrace, Resotran, and FerroTrace bottles. **b** Representative MPI images of 20 µg Fe (in 10 µL) of each magnetic tracer and their corresponding line profile. Data collection was repeated for *n* = 13 samples, prepared from two independent bottles per SPIO. **c** Relaxometry scans reveal differences in maximum signal amplitude and spatial resolution (full width at half maximum, FWHM) for magnetic tracers. Data are normalized to the quantity of SPIO (µg Fe). Data represent the mean of *n* = 3 technical replicates per SPIO. **d** Representative dynamic light scattering (DLS) intensity distributions for VivoTrax, Magtrace, and Resotran (*n* = 3).

MPI signal performance for VivoTrax, Magtrace, and Resotran was compared, with VivoTrax serving as the benchmark magnetic tracer (Fig. 1 and Table 2). Samples containing 20 µg Fe (in 10 µL) of each magnetic tracer (*n* = 13 samples per SPIO) were imaged by MPI (Fig. 1b and Table 2). Total (sum) MPI signal was significantly reduced for Resotran and FerroTrace compared with VivoTrax and Magtrace (*p* < 0.05); however, the magnitude of reduction was modest (~10%). Greater variability in the total (sum) MPI signal was observed for VivoTrax and Magtrace, potentially reflecting variation across two bottles.

**Table 2:** Summary of in vitro MPI characterization, relaxometry, and dynamic light scattering (DLS) results

Technique	Measurement	VivoTrax	Magtrace	Resotran	FerroTrace
MPI	Total (sum) signal per 20 µg Fe	2908 ± 368 a.u.	3009 ± 225 a.u.	2560 ± 60 a.u.	2650 ± 52 a.u.
MPI	Relative total (sum) signal	100 ± 12.7%	103 ± 7.7%	88 ± 2.1%	91 ± 1.8%
Relaxometry	Relative peak signal per µg Fe	100%	87.60%	77.70%	50.20%
Relaxometry	FWHM	10.4 mT	12.3 mT	10.6 mT	22.6 mT
DLS	Effective hydrodynamic diameter	57 ± 0.5 nm	46 ± 0.3 nm	56 ± 0.2 nm	Not measured
DLS	PDI	0.22	0.25	0.21	Not measured

Total (sum) MPI signal from *n* = 13 samples is reported in arbitrary units (a.u.) and as a percentage relative to VivoTrax. Relaxometry data are presented as peak signal relative to VivoTrax (%) and resolution estimated by full width half maximum (FWHM). Effective hydrodynamic diameter and average polydispersity index (PDI) were determined by DLS.

Relaxometry revealed significant differences in peak signal amplitude and spatial resolution among magnetic tracers (*n* = 3 per SPIO, Fig. 1c, Table 2). Peak signal normalized to SPIO quantity (µg Fe) was highest for VivoTrax, followed by Magtrace, then Resotran, with FerroTrace producing the lowest peak signal (all pairwise comparisons, *p* < 0.05). FWHM also differed across magnetic tracers, with VivoTrax demonstrating the narrowest profile and FerroTrace the broadest by approximately 2-fold. For a 5.7 T/m gradient (preclinical MPI), the estimated spatial resolution is 1.8 mm for VivoTrax, 2.2 mm for Magtrace, 1.9 mm for Resotran, and 4.0 mm for Ferrotrace, which provides high-resolution preclinical SLN imaging.

DLS measurements of effective hydrodynamic diameter and polydispersity index (PDI) for VivoTrax, Magtrace, and Resotran are summarized in Table 2. All three SPIO suspensions exhibited bimodal intensity distributions, consistent with mixed populations of single and clustered SPIOs, with PDIs ranging from 0.20 to 0.26. The overall hydrodynamic size distributions were similar across magnetic tracers. Representative DLS intensity profiles are shown in Fig. 1d (*n* = 3 per SPIO).

### In vivo MPI lymphography with Magtrace and Resotran

**Fig. 2:**
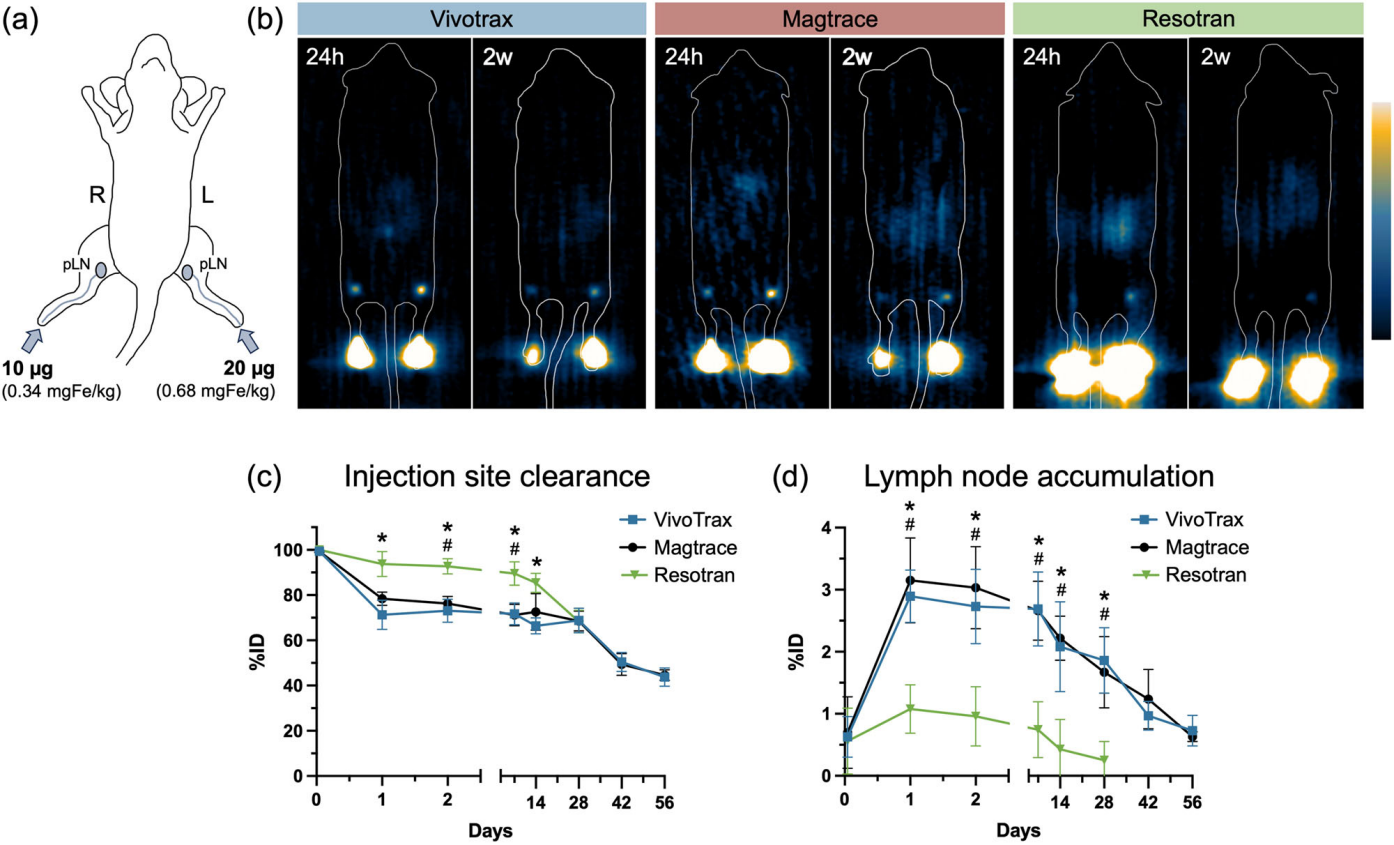
In vivo pharmacokinetics of VivoTrax, Magtrace, and Resotran to popliteal lymph nodes (pLN) following intradermal administration. **a** Mice received intradermal injections of SPIOs in the left (20 µg Fe, ~ 0.68 mg Fe/kg) and right (10 µg Fe, ~0.34 mg Fe/kg) footpads (*n* = 3 per magnetic tracer: VivoTrax, Magtrace, and Resotran). **b** Representative MPI for each magnetic tracer group at 1 day and 2 weeks post-injection show magnetic tracer localization at injection sites and draining pLNs. Refer to Figure S1 for additional imaging timepoints. **c** MPI quantification at footpads, expressed as percent injected dose (%ID), demonstrates gradual clearance of SPIOs from the injection site. On average, 70% of the MPI signal remains after 4 weeks, indicating long-term retention of each SPIO. **d** MPI quantification at draining pLNs expressed as %ID shows uptake within the first day, followed by a persistent but declining signal over time. Data were pooled across dose groups (see also Figure S2). The symbol * indicates timepoints with *p* < 0.05 for VivoTrax vs. Resotran, and # represents *p* < 0.05 Magtrace vs. Resotran. There were no statistical differences between VivoTrax and Magtrace at any timepoint.

We demonstrated in vivo MPI lymphography using VivoTrax, Magtrace and Resotran after injecting two doses of each SPIO into both hind footpads (Fig. 2a). MPI signal was detected at both the injection site and in the draining pLN as early as 1-hour post-injection, with peak signal observed at 1 day for all magnetic tracers, and signals persisted at least 4-8 weeks (Fig. 2b and Figure S1).

Quantitative pharmacokinetics demonstrated progressive MPI signal clearance from the injection site (Fig. 2c) with corresponding accumulation in the pLN (Fig. 2d). Doubling the administered iron dose, from 10 µg Fe in the right footpad to 20 µg in the left footpad, produced an approximately two-fold increase in MPI signal in the corresponding ipsilateral pLN (Figure S2a). Figure 2c, d include pooled %ID data for both dose groups, which was justified by the repeated-measures ANOVA showing no significant effect of the dose on %ID over time, determined for both the injection site and pLN (*p* > 0.05) (Figure S2b).

Magtrace and VivoTrax exhibited comparable injection site clearance and pLN uptake kinetics, each reaching ~3%ID at 1 day before gradually declining (Fig. 2c, d). Resotran reached ~1%ID in pLNs at 1 day. Clearance of Resotran from the injection site was slower during the first 2 weeks compared to Magtrace and VivoTrax (*p* < 0.05). No significant differences in %ID at pLNs were observed between VivoTrax and Magtrace at any timepoint, whereas both magnetic tracers demonstrated over 2.7× higher uptake on average than Resotran from 1 day onwards (*p* < 0.05). Mice with VivoTrax and Magtrace were imaged for up to 8 weeks, whereas Resotran was imaged for up to 4 weeks because the pLN MPI signal declined earlier. Direct comparisons of magnetic tracers in the same mouse (VivoTrax vs. Resotran and Magtrace vs. Resotran) further confirmed these results (Figure S3).

### Ex vivo verification

**Fig. 3:**
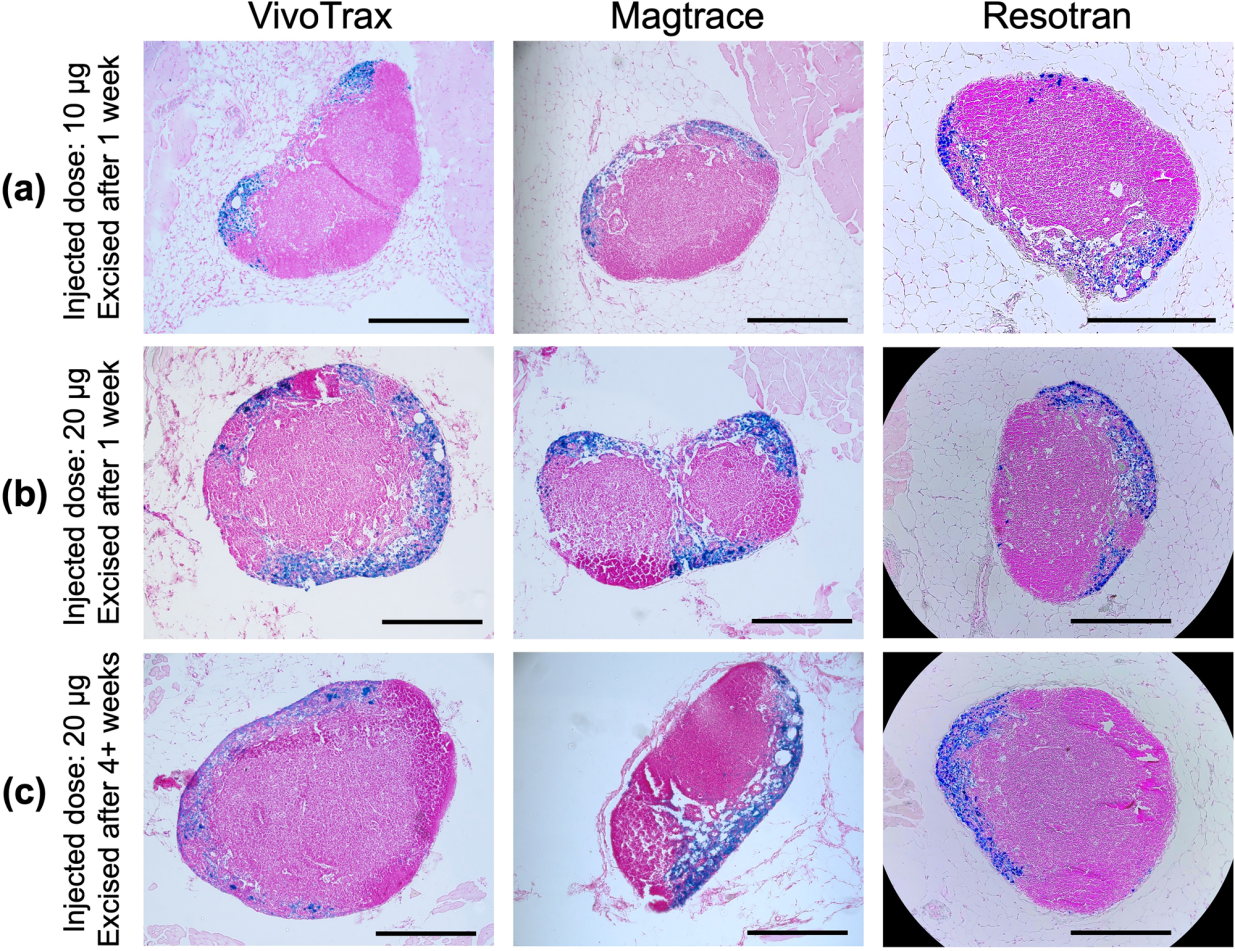
Perls’ Prussian Blue (PPB) staining verifies iron localization in pLNs following VivoTrax, Magtrace, and Resotran injection into hind footpads. For each magnetic tracer, lymph nodes were collected 1 week after (**a**) 10 µg Fe or (**b**) 20 µg Fe in the ipsilateral footpad. **c** At endpoint, pLNs were collected at 8 weeks (VivoTrax, Magtrace) or 4 weeks (Resotran) to verify MPI signals. Scale bars = 400 µm.

PPB staining confirmed iron deposition in pLNs following injection of VivoTrax, Magtrace, and Resotran (Fig. 3). Iron staining was identified at the periphery of all lymph nodes in the subcapsular sinus, the initial site where lymph enters via afferent lymphatics. All magnetic tracers showed PPB staining at 1 week, with staining persisting to 8 weeks for VivoTrax and Magtrace and at least 4 weeks for Resotran (at endpoint).

### FerroTrace for MPI

**Fig. 4:**
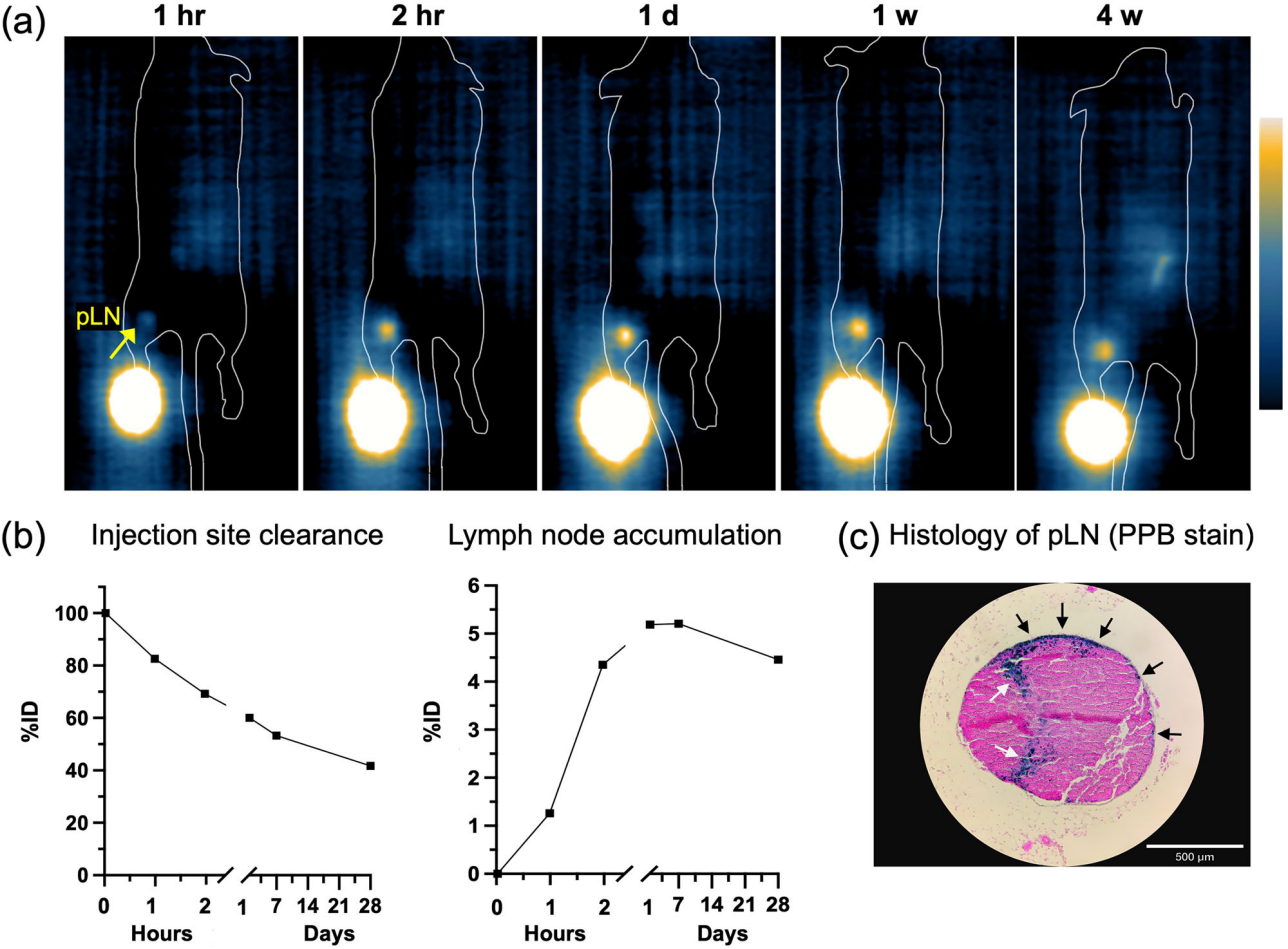
FerroTrace for MPI lymphography (*n* = 2). **a** Longitudinal MPI acquired at 1 h, 2 h, 1 day, 1 week, and 4 weeks following injection of 20 µg Fe FerroTrace into the left hind footpad. 2D MPI projections were reconstructed using the X-space formulation. **b** Quantification of MPI signal demonstrated progressive clearance from the injection site, with 42%ID remaining at the study endpoint, and accumulation within the draining pLN, reaching a maximum of 5.2%ID at 1 day. **c** Perls’ Prussian Blue (PPB) staining confirmed iron deposition in the pLN 4 weeks post-injection, predominantly localized to the subcapsular space (black arrows) and trabecular sinuses (white arrows).

Next, we assessed the performance of FerroTrace, an investigational SPIO designed for maximal retention in SLNs by targeting the mannose receptor CD206. Following intradermal administration, FerroTrace demonstrated clear lymphatic drainage to the pLN, with MPI signal detected by 1-hour post-injection (*n* = 2) (Fig. 4). Lymph node signal peaked after 1 day (5.2%ID) and remained high for at least 4 weeks, indicating stable long-term SPIO retention. At the injection site, MPI signal declined to ~40% by 4 weeks (Fig. 4b). PPB staining was verified in draining pLN 4 weeks after intradermal injection of FerroTrace, with localization in the subcapsular sinus as well as deeper penetration into lymph nodes via trabecular sinuses. These feasibility results suggest that FerroTrace can be transported to the draining pLN and provide persistent MPI contrast suitable for lymphography.

Following intravenous administration of FerroTrace in the mouse bearing a 4T1 mammary tumor, in vivo MPI showed signal in the liver and spleen, but no signal was detected in the tumor region (Figure S4). Ex vivo MPI of the excised tumor revealed a low MPI signal, equivalent to 3.0 µg Fe FerroTrace ( ~ 0.25%ID). PPB confirmed the presence of iron within the tumor. Since this experiment was performed in a single mouse, these findings should be interpreted as preliminary observations.

### Clinical-scale MPI with Magtrace

**Fig. 5:**
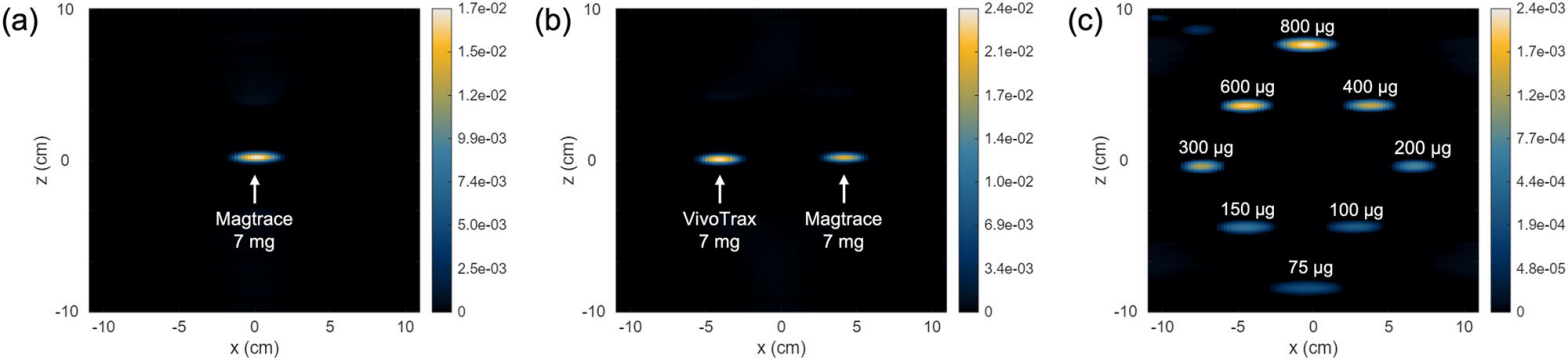
Magtrace detection on a clinical-scale MPI system. **a** Magtrace sample containing 7 mg Fe in 0.25 mL. **b** Direct comparison of Magtrace and VivoTrax samples, each containing 7 mg Fe in 0.25 mL. **c** Dilution series of 8 Magtrace samples containing 75–800 µg Fe in 100 µL total volume. Image (**c**) is displayed with non-linear scaling (image values raised to the power of 0.5) to aid visualization of the full signal range. Image values are in arbitrary units (a.u.).

Magtrace produced clear MPI signal when imaged on the clinical-scale MPI system, confirming compatibility with clinical-scale imaging hardware (Fig. 5a). Clinical-scale spatial resolution was estimated from line profiles of the imaged Magtrace (Figure S5). In direct side-by-side comparison at matched iron mass (7 mg Fe in 0.25 mL), both Magtrace and VivoTrax were detected, with a higher peak MPI signal observed for VivoTrax (Fig. 5b). Magtrace was also detected across a dilution series spanning 75–800 µg Fe in 100 µL (Fig. 5c), with peak MPI signal scaling with iron mass.

### Human-scale SLN phantom

**Fig. 6:**
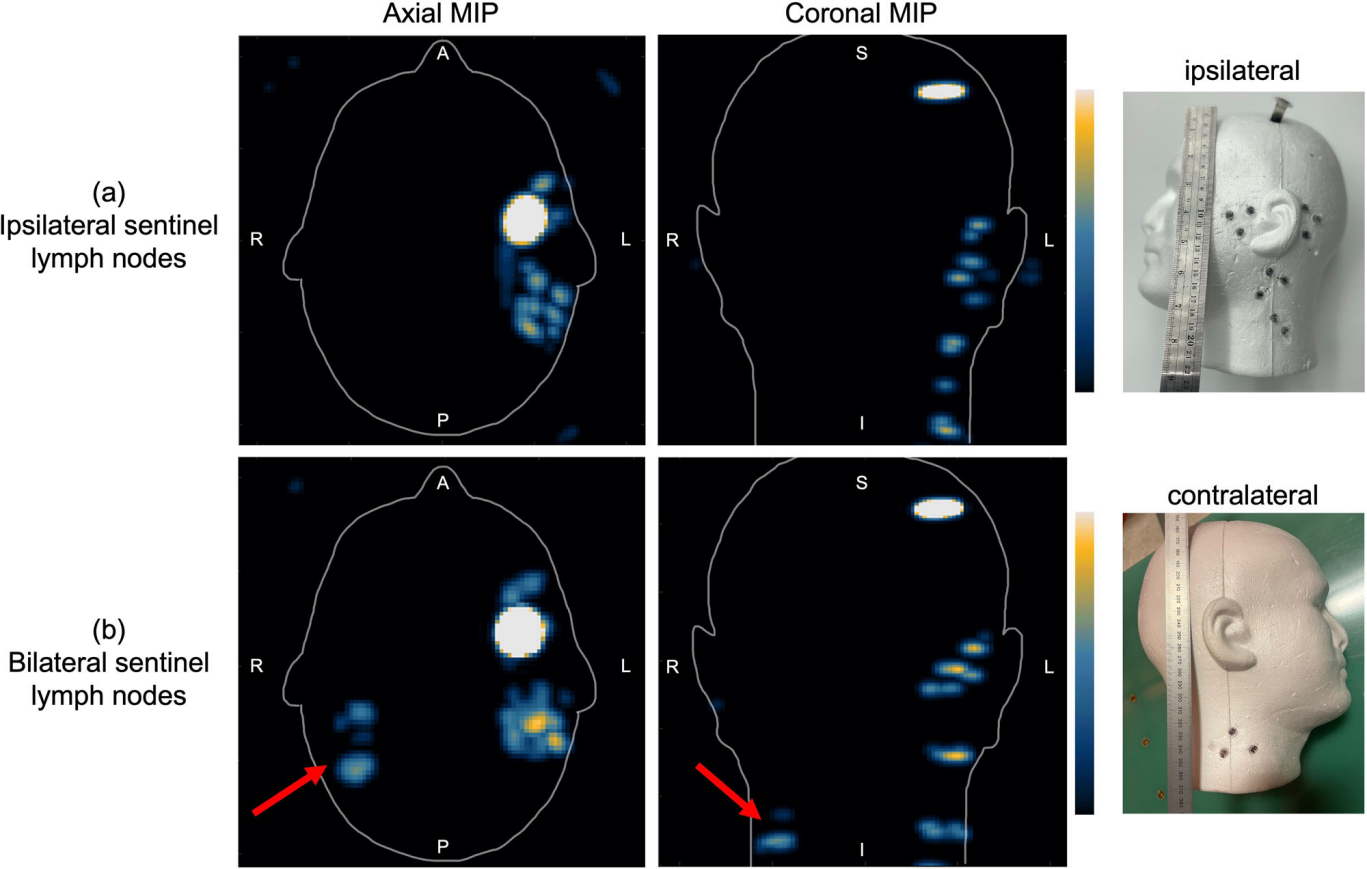
Human-scale MPI phantoms modeling ipsilateral vs. bilateral lymphatic drainage patterns. **a** Ipsilateral phantom consists of a vial of 7 mg Fe to model an injection dose and 11 mock draining lymph nodes in the periauricular and cervical regions on the ipsilateral side (100 µg Fe in 100 µL). The axial and coronal MIPs show MPI signals only on the ipsilateral side. **b** Bilateral phantom has an additional 3 mock lymph nodes on the contralateral side. These contralateral signals are detected in axial and coronal MIPs (red arrows).

Using a human-scale MPI system, we imaged a human-sized head phantom designed to model scalp drainage to periauricular and cervical lymph nodes with VivoTrax. In the ipsilateral phantom configuration (Fig. 6a), axial and coronal MIPs showed the 11 lymph node phantoms on the ipsilateral side, with no detectable MPI signal on the contralateral side, demonstrating high spatial specificity. In the bilateral configuration, MPI signal from the three contralateral lymph node phantoms was also visualized in both axial and coronal MIPs (Fig. 6b). These results demonstrate that the clinical-scale MPI system can be used to distinguish ipsilateral versus bilateral drainage patterns, while detecting multiple lymphatic basins within a clinically relevant imaging field of view.

## Discussion

The clinical translation of MPI has in part been constrained by the limited availability of clinical magnetic tracers with suitable MPI properties. Here, we evaluated three SPIOs with established or emerging clinical applicability—Magtrace, Resotran, and FerroTrace—selected based on prior human use and regulatory status^15,36,51^. Through a stepwise investigation encompassing in vitro MPI characterization, in vivo murine lymphography to define pharmacokinetics, and human-scale phantom imaging to assess clinical feasibility, these magnetic tracers demonstrated high compatibility with MPI detection and lymphatic mapping.

We provide the first evaluation of Magtrace and FerroTrace for MPI against the established preclinical benchmark VivoTrax^52^. Total (sum) MPI signal did not significantly differ between Magtrace and VivoTrax (*p* > 0.05, Table 2); however, VivoTrax produced modestly higher peak signal in relaxometry (by ~14%), as also visualized in images acquired on both preclinical and clinical-scale MPI systems (Figs. 1b and 5b). In our opinion, this modest difference in peak signal is unlikely to hinder the practical translation of Magtrace for MPI. Resotran showed approximately 9% reduction in total (sum) MPI signal compared with VivoTrax (*p* < 0.05, Table 2), while prior reports have described its in vitro performance within 10% of Resovist (ferucarbotran)^37,38^. DLS measurements were consistent with the manufacturer’s reports and revealed bimodal size distributions across VivoTrax, Magtrace, and Resotran, suggesting similarities in core structure, including single and clustered SPIOs. Small differences in hydrodynamic diameter may be related to the different SPIO formulations and stability over time and across bottles. FerroTrace produced significantly lower MPI signal compared to VivoTrax (*p* < 0.05, Tables 2), and 2-fold reduced spatial resolution in relaxometry, likely related to differences in core composition^24^. Collectively, these results indicate that Magtrace, Resotran, and FerroTrace are compatible with MPI. Historically, VivoTrax has served as a research benchmark for MPI, but it is no longer commercially available. The observed performance of Magtrace and Resotran supports their use as suitable magnetic tracers for future in vitro benchmarking in MPI.

In vivo, all SPIOs demonstrated effective lymphatic transport to the primary draining pLN, supporting their use for SLN imaging. Magtrace and VivoTrax showed no significant differences in injection site clearance and lymph node uptake kinetics by %ID, which were consistent with prior reports and passive interstitial transport^53,54^. However, Resotran showed significantly lower %ID in pLNs and slower clearance from the site of injection, which may be related to its formulation (Table 1). FerroTrace demonstrated robust lymph node accumulation and excellent retention over several weeks despite having a lower peak signal by relaxometry, indicating that favorable pharmacokinetics can compensate for reduced magnetic performance. The dual transport mechanisms proposed for FerroTrace, including passive interstitial drainage coupled with macrophage-targeting, may contribute to the enhanced lymph node uptake^24^. Collectively, these findings indicate that magnetic tracer suitability for MPI lymphography depends on in vivo pharmacokinetics, which cannot be predicted by in vitro MPI performance alone.

Iron deposition in mouse draining pLNs scaled proportionally with injected dose across Magtrace, Resotran, and VivoTrax. Doubling the administered iron produced an approximately two-fold increase in MPI signal, while the percent injected dose (or utilization rate^55^) remained consistent across dose levels. Prior studies have shown that at higher doses, lymph node iron uptake reaches a ‘saturation limit’^54,55^. The proportional uptake observed here suggests that dosing remained within the pre-saturation range and supports the feasibility of adjustable dosing strategies, where higher doses may be used to prolong SLN detectability for extended workflows. Notably, the clinical evolution of Magtrace dosing, from an initial 2 mL approval to inclusion of a 1 mL option (PMA P160053, S009), reflects efforts to minimize skin staining and MRI artifacts^56^. Ultra-low doses have also been explored to label SLNs in melanoma^17^. Collectively, these findings suggest that magnetic tracer dosing may be optimized according to procedural considerations.

Lymph node labeling in mice persisted for extended durations following SPIO administration. Prior studies with other preclinical SPIOs show MPI signal in lymph nodes for 4-8 days^30,31^. In this study, the MPI signal remained detectable for at least four weeks, and we imaged up to eight weeks for Magtrace and VivoTrax. VivoTrax, Magtrace, and Resotran accumulate within the subcapsular sinus, close to the afferent lymphatic entry point, whereas FerroTrace additionally exhibits localization extending into trabecular sinuses^24^. Such differences may be significant for SLN labeling, and may influence SPIO retention, flow to echelon nodes, and the durability of the MPI signal. Altogether, prolonged retention of SPIOs over weeks extends the demonstrated time window for magnetic SLNB compared with conventional radiotracer workflows, which are constrained by isotope decay and same- or next-day coordination between injection, imaging, and surgery. Magnetic tracers may, therefore, be particularly valuable in workflows where same-day coordination is impractical, for example, in oral cavity cancers where SPIOs are already being adopted in combination with magnetometers and MRI (Table 3). This long-term retention of SPIOs also supports new workflows involving tracer administration days to weeks in advance of surgery, such as delayed SLNB^57–60^, pre-labeling of lymphatic pathways prior to therapies that may disrupt normal drainage patterns^61^, and post-operative SLN detection^62^.

**Table 3:** Summary of clinical studies reporting or proposing the use of SPIOs for SLN mapping using magnetometer and MRI in oral cavity cancers.

Reference	Title	Magnetic Tracer	Dose	*n*
^87^	Superparamagnetic iron oxide-enhanced interstitial magnetic resonance lymphography to detect a sentinel lymph node in tongue cancer patients	Resovist	2.8–8.4 mg Fe (0.1–0.3 mL)	3
^22^	Magnetic detection of sentinel nodes in oral squamous cell carcinoma by means of superparamagnetic iron oxide contrast	Magtrace	56 mg Fe (2 mL)	11
^21^	A complete magnetic sentinel lymph node biopsy procedure in oral cancer patients: A pilot study	Sienna+	11.2–22.4 mg Fe (0.4–0.8 mL)	10
^70^	A comprehensive grading system for a magnetic sentinel lymph node biopsy procedure in head and neck cancer patients	Sienna+	11.2–22.4 mg Fe (0.4–0.8 mL)	8
^88^	Sentinel lymph node biopsy with a handheld cordless magnetic probe following preoperative MR lymphography using superparamagnetic iron oxide for clinically N0 early oral cancer: A feasibility study.	Resovist	44.6 mg Fe (1.6 mL)	27
^27^	Mannose-labelled magnetic nanoparticles for sentinel lymph node biopsy in oral squamous cell carcinoma: Initial results from a phase 1 clinical trial	FerroTrace and Magtrace	10–28 mg Fe	8
^23^	Magnetic sentinel lymph node detection using superparamagnetic iron oxide in early-stage oral squamous cell carcinoma: design and rationale of the multicenter magnetics trial - study protocol	Magtrace	11.2 mg Fe (0.4 mL)	82

Although magnetic tracers are increasingly adopted for SLNB, surgeons lack a preoperative lymphatic map when relying solely on magnetometer-based detection. This concern is less pronounced in breast cancer, where lymphatic drainage typically follows a predictable path to the axilla. Indeed, studies have demonstrated no significant effect of preoperative imaging on intraoperative SLN detection rates in breast cancer^63^ and the Magtrace–Sentimag system approved in breast cancer does not require preoperative imaging^15^. However, preoperative imaging is critical for SLN mapping in cancers with complex lymphatic anatomy. In melanoma, tumors may arise from any site on the body and can drain to single or multiple nodal basins through unpredictable lymphatic pathways^4,28^. Imaging therefore, plays an essential role in identifying which basin(s) should be prepared for surgery and interrogated in melanoma patients. Magnetic techniques have been explored in melanoma and demonstrated non-inferiority to radioisotope-based approaches in the MELAMAG trial; however, lymphoscintigraphy was still required to define the nodal basins preoperatively^20^. More recent studies have incorporated MRI to localize SLNs prior to magnetic probe–guided surgery^17,64^. Similarly, studies in prostate cancer^62^ and oral cavity cancer (Table 3) have relied on MRI to assess SLN distribution after SPIO injection. However, MRI detection of SPIOs depends on negative contrast, which can render SLN identification ambiguous and often requires baseline imaging^17,64^ and dynamic imaging to distinguish first-echelon from downstream nodes^25,27^. MPI addresses this limitation by providing unambiguous “hot-spot” contrast with direct magnetic tracer quantification, potentially supporting SLN identification using established criteria such as the 10% rule^65^.

In head and neck cancer, SLN mapping enables targeted excision of lymph nodes draining a tumor site, reducing the morbidity associated with total elective lymph node dissection^1–5^. SLN mapping in head and neck cancer is also being explored for tailoring treatments in radiation oncology (NCT05451004, NCT06706401)^5–8,66–68^. SPIOs are being tested for SLN mapping in head and neck cancer using magnetometer detection and MRI (Table 3). Because a key clinical question in head and neck cancer is whether tumor-draining lymphatics cross the midline, we constructed a human-scale phantom incorporating ipsilateral and bilateral drainage configurations to evaluate MPI for lymphatic mapping tasks. Both configurations were successfully imaged, demonstrating that contralateral nodal involvement can be identified within a human head-sized imaging field of view. The phantom incorporated 14 lymph node targets, approximating the nodal burden encountered in clinical head and neck dissections, where retrieval of ~15–20 SLNs is typical^69^. The clinical-scale MPI scanner resolved VivoTrax samples separated by 1.5–2 cm and detected iron quantities consistent with reported SPIO deposition in SLNs (~100 µg)^70–72^. Importantly, Magtrace also produced clear signal on the clinical-scale MPI system and remained detectable across a dilution series spanning 75–800 µg Fe in 100 µL, representing as low as 1.3% of clinically approved SPIO doses (56 mg), supporting the feasibility for detecting Magtrace deposition in SLNs with clinical-scale MPI^70^. Collectively, these findings verify the technical feasibility of MPI for preoperative SLN mapping, with potential to support precise surgical and radiation planning to avoid unnecessary bilateral intervention in the majority of patients with head and neck cancer. MPI could play a complementary role by providing surgeons with preoperative images showing the location of SPIO-labeled SLNs, while handheld magnetometers provide numerical counts to guide intraoperative node localization.

Beyond lymphatic imaging, SPIOs have been used for a wide range of applications, including cell tracking^73^, interventional imaging^74^, and imaging inflammation^75^, underscoring their potential as a versatile imaging agent. In this study, we explored FerroTrace as a candidate for tumor-associated macrophage imaging by MPI, leveraging its proposed CD206-mediated targeting mechanism. MPI detected tumor accumulation (~0.25% ID) ex vivo; however, in vivo tumor visualization was not achieved, and uptake measured ex vivo was lower than levels typically reported for nanoparticle-based tumor targeting^76^. Future work should involve testing in larger cohorts and optimizing blood circulation half-life to improve macrophage uptake, through strategies such as tuning core size and surface modifications^47,77,78^.

In this work, we evaluated Magtrace, Resotran, and FerroTrace, expanding the portfolio of magnetic tracers compatible with MPI and human use. Leveraging SPIOs with established safety profiles may represent the most efficient and practical path toward human MPI studies. Resotran has already been used in human cadaver MPI studies, demonstrating feasibility for interventional imaging^74^. Continued integration of these magnetic tracers into preclinical MPI investigations will strengthen the translational bridge towards clinical application. In parallel, continued development of next-generation nanoparticles specifically optimized for MPI signal performance offers substantial long-term potential to enhance resolution, sensitivity, and biologically targeted imaging applications^79,80^.

Magtrace, Resotran, and FerroTrace enabled quantitative murine MPI lymphography with specific detection of the primary draining lymph node, representing the first in vivo MPI studies performed with these clinical SPIOs. Lymph nodes were detected over extended timeframes (4–8 weeks), expanding opportunities for flexible SLNB workflows beyond the constraints of radiotracer half-life. Aligned with the growing use of SPIOs for SLN mapping in head and neck cancer, our human-scale MPI phantom positions MPI to identify cases with bilateral lymph node involvement and support complex SLN mapping tasks.

## Methods

### SPIO characterization for MPI

Four SPIO formulations were evaluated for MPI: VivoTrax (5.5 mg Fe/mL, Magnetic Insight Inc., Alameda, CA), Magtrace (28 mg Fe/mL, Endomagnetics, Cambridge, UK), Resotran (28 mg Fe/mL, B.E. imaging GmbH, Germany), and FerroTrace (56 mg Fe/mL, Ferronova, Australia). Sample preparation was based on manufacturer-reported iron concentrations for each SPIO. MPI signal characteristics were assessed by preparing 20 µg Fe for each magnetic tracer, each diluted in a total volume of 10 µL phosphate buffered saline (PBS) (20 µg/10 µL). For each SPIO, 13 samples were prepared and imaged by MPI across three separate days. For VivoTrax, Magtrace, and Resotran, samples were prepared from two independent bottles.

MPI 2D projection scans were acquired on a MOMENTUM^**T**M^ MPI scanner (Magnetic Insight Inc.) using a 12 × 6 × 6 cm^3^ field of view, x- and z-axis transmit channels, a 5.7 T/m gradient, and radiofrequency amplitudes of 20 mT (*x*) and 23 mT (*z*). Each scan was acquired over 2 min and reconstructed using X-space formulation with an Equalization filter^81^.

Magnetic particle relaxometry was performed using the RELAX acquisition on the Momentum MPI scanner, with a 20 mT drive field and 160 mT bias field. Samples of VivoTrax, Resotran, and Magtrace contained 28 µg Fe (in 10 µL), while FerroTrace contained 44 µg Fe (in 10 µL), with iron masses selected to yield peak signals of ~1 a.u. All samples were positioned at *x* = 0 for RELAX measurement. Three replicate samples were scanned then averaged to generate the point spread function for each SPIO. Lastly, the relaxometry data were normalized by the SPIO mass to compare signal per µg Fe.

Hydrodynamic diameters were measured by dynamic light scattering (DLS) using a Brookhaven NanoBrook Omni system (Holtsville, NY). Nanoparticle formulations were diluted in PBS and analyzed in triplicate for Magtrace, Resotran, and VivoTrax.

### Animal studies

Healthy male C57BL/6 mice (20 weeks old; 28–30 g; Charles River, Canada or USA) were used for in vivo lymphography studies. Seven mice were obtained and housed at the Robarts Research Institute (RRI), where all procedures were conducted under a protocol approved by the Western University Council on Animal Care. An additional six C57BL/6 male mice were obtained at Magnetic Insight Inc. under a protocol approved by an Institutional Animal Care and Use Committee provided by In Vivo Strategies. One female BALB/c mouse (13 weeks old; Charles River, Canada) was also included for preliminary studies on tumor-associated macrophages, performed at RRI under a protocol approved by the Western University Council on Animal Care. All animals were maintained under standard housing conditions with ad libitum access to food and water. During the injection and imaging procedures, mice were anesthetized under 2% isoflurane in 100% oxygen. At the study endpoint, mice were euthanized by isoflurane overdose prior to tissue collection, in accordance with institutional animal care guidelines.

### In vivo MPI lymphography

Mice received intradermal injections of magnetic tracers into the hind footpads. The ipsilateral popliteal lymph node (pLN), the first draining lymph node from the injection site, was designated as the surrogate SLN in healthy mice to evaluate lymphatic transport in the absence of tumor-induced lymphatic remodeling. A dose of 20 µg Fe (~0.68 mg Fe/kg) was injected into the left footpad and 10 µg Fe (~0.34 mg Fe/kg) into the right footpad, with each injection delivered in a total 30 µL suspension of PBS using a 30-gauge needle. These doses were selected by body-weight scaling from SPIO doses used for human SLNB, with 20 µg Fe approximating the 56 mg Fe dose used for Magtrace and FerroTrace^15,51^, and 10 µg Fe approximating the 28 mg Fe reduced-dose option for Magtrace (PMA P160053, S009).

For VivoTrax, Magtrace, and Resotran, three mice per magnetic tracer were included. To enable direct, within-animal comparisons between SPIOs, two additional mice received paired bilateral injections: one mouse received Resotran in the left footpad and VivoTrax in the right footpad, while another received Magtrace in the left and Resotran in the right footpad, each at 20 µg Fe. Mice underwent serial 3D MPI (35 projections, scan time = 35 min) at 10 min, 24 h, 48 h, 1 week, 2 weeks, and 4 weeks post-injection, with extended imaging at 6 and 8 weeks performed for mice with Magtrace and VivoTrax. For FerroTrace studies, mice received 20 µg Fe intradermally into the left hind footpad (*n* = 2) and were imaged repeatedly using 2D MPI projections over 4 weeks.

### Image analysis and quantification

The MPI signal at the injection site, and pLN was quantified over time to characterize lymphatic transport for each SPIO formulation. MPI datasets were reconstructed using the X-space algorithm and 3D images were reconstructed by combining 35 projections using Filtered Back Projection. For visualization, select images were reconstructed using a model-based algorithm^82,83^. An optical reference photograph acquired using the integrated camera on the MPI system was used to manually outline mouse anatomy.

For MPI signal quantification in vitro and in vivo, a region of interest (ROI) was defined using a threshold of ≥0.5 × maximum value^84^. Total MPI signal was calculated as the sum of the intensities of all voxels within each ROI. For in vivo data, a percent injected dose (%ID) was calculated for signals in lymph nodes and injection sites for each timepoint (Eq. 1). The baseline signal was the summation of the total MPI signal at the first imaging timepoint (10 minutes) and included signal at the injection site and ipsilateral pLN, if present.

1 $$\% {\rm{ID}}=({\rm{pLN}}\; {\rm{signal}}/{\rm{baseline}}\; {\rm{signal}}){{\times}}100 \%$$

### Histology

To verify MPI signal ex vivo, pLNs were excised for histology following euthanasia at the final imaging timepoint, including one mouse from the VivoTrax, Magtrace, and Resotran cohorts at 1 week. Endpoint histology was conducted to verify residual MPI corresponded to persistent iron deposition in lymph nodes. pLNs were fixed in 4% paraformaldehyde for 12 hours, then transferred to 70% ethanol. Fixed lymph nodes were paraffin-embedded and sectioned as pairs of 5 µm sections (RRI Molecular Pathology Department; or Zyagen Histology Services, San Diego, CA), then stained with Perls’ Prussian blue (PPB) with nuclear fast red counterstain (ab150674, Abcam, Waltham, MA). Microscopy was performed using the Echo 4 Revolve Microscope with a 20× objective lens (CA, USA).

### Exploratory intravenous FerroTrace imaging in a tumor-bearing mouse

A pilot experiment was performed to explore the potential of FerroTrace for in vivo imaging of tumor-associated macrophages in a breast cancer model. Although FerroTrace is under clinical investigation as a peritumorally injected magnetic tracer for SLN mapping, this experiment tested whether intravenous administration could exploit its proposed mannose receptor CD206-targeting mechanism for macrophage labeling in situ. 4T1 murine breast cancer cells (3 × 10⁵ cells in 30 µL PBS) were implanted into the fourth (inguinal) mammary fat pad of a female BALB/c mouse. After 19 days of tumor growth, this mouse received 1.2 mg FerroTrace intravenously via the tail vein (100 µL), administered 48 h prior to 3D MPI. Following imaging, the tumor was excised and imaged ex vivo with 2D MPI alongside 9 calibration samples (1.1–6.3 µg Fe in 1 mL) to estimate iron mass in the tumor. After imaging, the tumor was processed for PPB staining to confirm iron localization.

### Clinical-scale MPI with Magtrace

Further SPIO characterization was conducted on a clinical-scale MPI system. Magtrace and VivoTrax samples containing 7 mg Fe (in 0.25 mL total volume) were imaged for direct comparison of MPI signal. A sensitivity analysis was performed by imaging Magtrace samples containing 75–800 µg Fe (in 100 µL total volume) to assess the detectable iron range.

Imaging was performed on a prototype clinical-scale MPI system (Magnetic Insight Inc.) with a 60 cm magnet-free bore operating in field-free point (FFP) mode, acquiring a 22 × 22 × 20 cm³ field of view with 0.28 × 0.28 × 0.55 T/m selection-field gradients. Images acquired on the clinical-scale system were reconstructed using multi-harmonic gridded 3D deconvolution (MH3D) algorithm^85^.

### Human-scale SLN phantom

A human-scale head-and-neck phantom was constructed using a Styrofoam head with Eppendorf tubes positioned along scalp drainage pathways into periauricular and cervical lymph node regions. A 1.5 mL Eppendorf tube filled with 7 mg VivoTrax was placed at the vertex to represent a scalp injection for melanoma. Eleven 0.2 mL Eppendorf tubes were placed in ipsilateral periauricular and cervical lymph node positions, and three additional tubes were added contralaterally for the bilateral configuration. All lymph node tubes contained 100 µg Fe VivoTrax in 100 µL. Imaging was similarly performed on the clinical-scale MPI system using a 22 × 22 × 24 cm^3^ field of view.

### Statistics

Statistical analyses were performed using GraphPad Prism (version 10.5.0; GraphPad Software, San Diego, CA). For in vitro comparisons of MPI signal across magnetic tracers, a one-way analysis of variance (ANOVA) was used with Tukey’s multiple comparisons test for post hoc analyses. For longitudinal in vivo lymphography data, injection site %ID and pLN %ID were analyzed separately. For each tracer, the effect of dose on %ID was evaluated using two-way repeated-measures ANOVA, with dose and time as factors and repeated measurements collected over time. Because dose did not significantly affect %ID, the 10 µg Fe and 20 µg Fe dose groups were pooled for subsequent pharmacokinetic comparisons. Differences in %ID among VivoTrax, Magtrace, and Resotran were then evaluated using two-way repeated-measures ANOVA, with tracer and time as factors. Tukey’s multiple comparisons test was used for post hoc pairwise comparisons. A *p*-value of 0.05 was used to determine statistical significance. Data are presented as mean ± standard deviation.

## Supplementary information

Supplementary Information

## Data Availability

All data generated or analysed during this study are included in this published article and its supplementary information files.
