# Supplementary material for: Magnetic particle imaging tracers for human applications: preclinical in vivo evaluation of Magtrace, Resotran, and FerroTrace
Source: Npj Imaging. 2026 Jul 21;4:48. doi: 10.1038/s44303-026-00182-7 (PMC13388925; doi:10.1038/s44303-026-00182-7)
Supplement: Supplementary file 1 — Supplementary Information [file 44303_2026_182_MOESM1_ESM.pdf]

Supplementary Materials

Magnetic particle imaging tracers for human applications: preclinical *in vivo* evaluation of Magtrace, Resotran, and FerroTrace

Olivia C. Sehl[1,2*], Nitara Fernando[2], Petrina Kim[1], A. Rahman Mohtasebzadeh[1], Toby Sanders[1], Kelvin Guo[1], Benjamin Fellows[1], Michael D. Alvarado[3], Benjamin Thierry[4], Marcela Weyhmiller[1], Joan M. Greve[1], Stephen Y. Lai[5], Patrick W. Goodwill[1], Paula J. Foster[2]

* Corresponding Author (email: osehl@uwo.ca)

1. Magnetic Insight Inc, Alameda, CA, USA
2. Department of Medical Biophysics, Western University, Robarts Research Institute, London, ON, Canada
3. Department of Surgery, University of California San Francisco, San Francisco, CA, USA
4. Future Industries Institute, Adelaide University, Adelaide, Australia
5. Department of Head and Neck Surgery, University of Texas MD Anderson Cancer Center, Houston, TX, USA

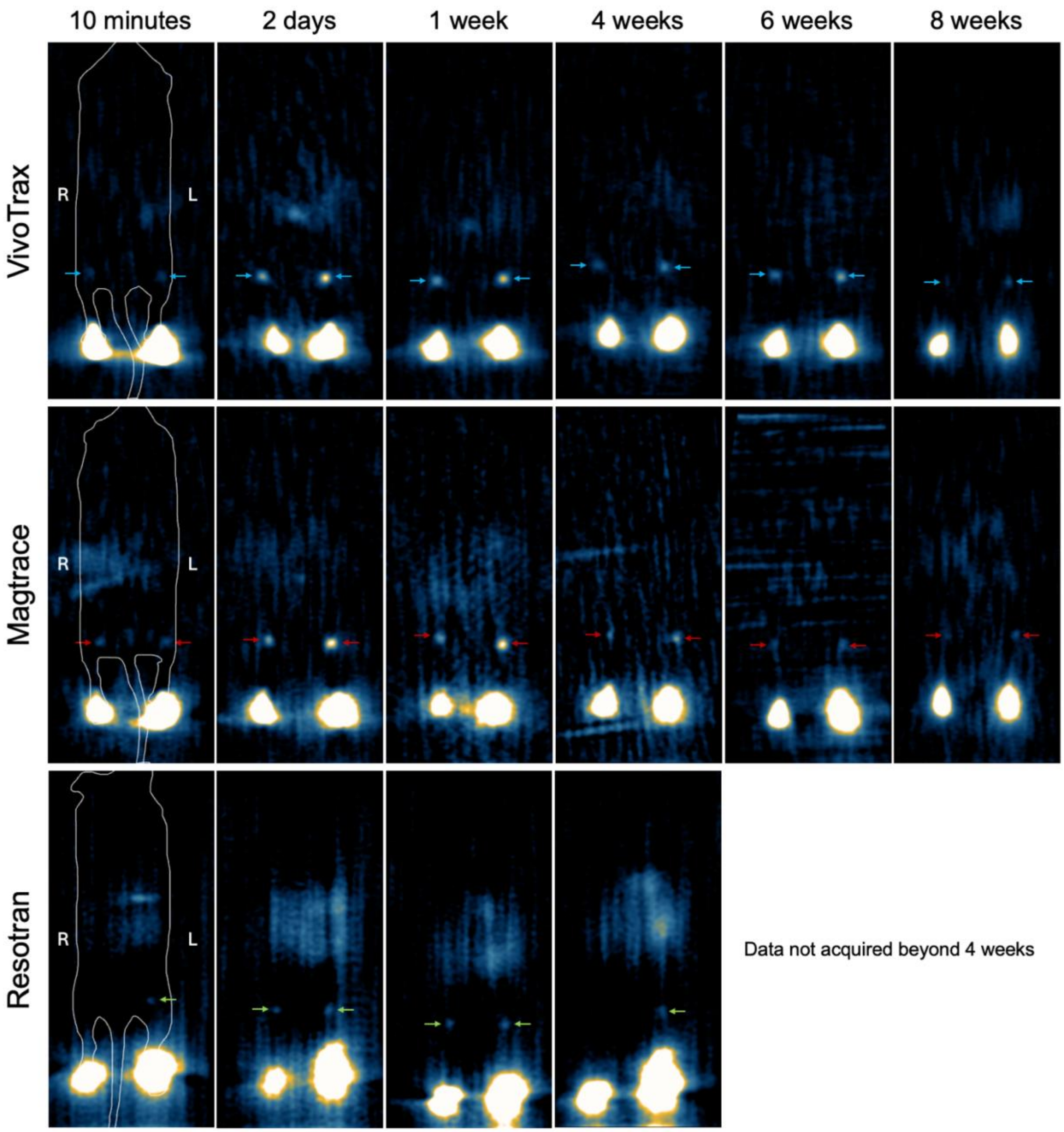

Figure S1. Additional timepoints for *in vivo* lymphography with VivoTrax, Magtrace, and Resotran after 10 minutes, 2 days, 1 week, 4 weeks, 6 weeks, and 8 weeks. Arrows denote draining popliteal lymph nodes. The left (L) footpad received 20 µg Fe and the right (R) footpad received 10 µg Fe. Refer also to Figure 2.

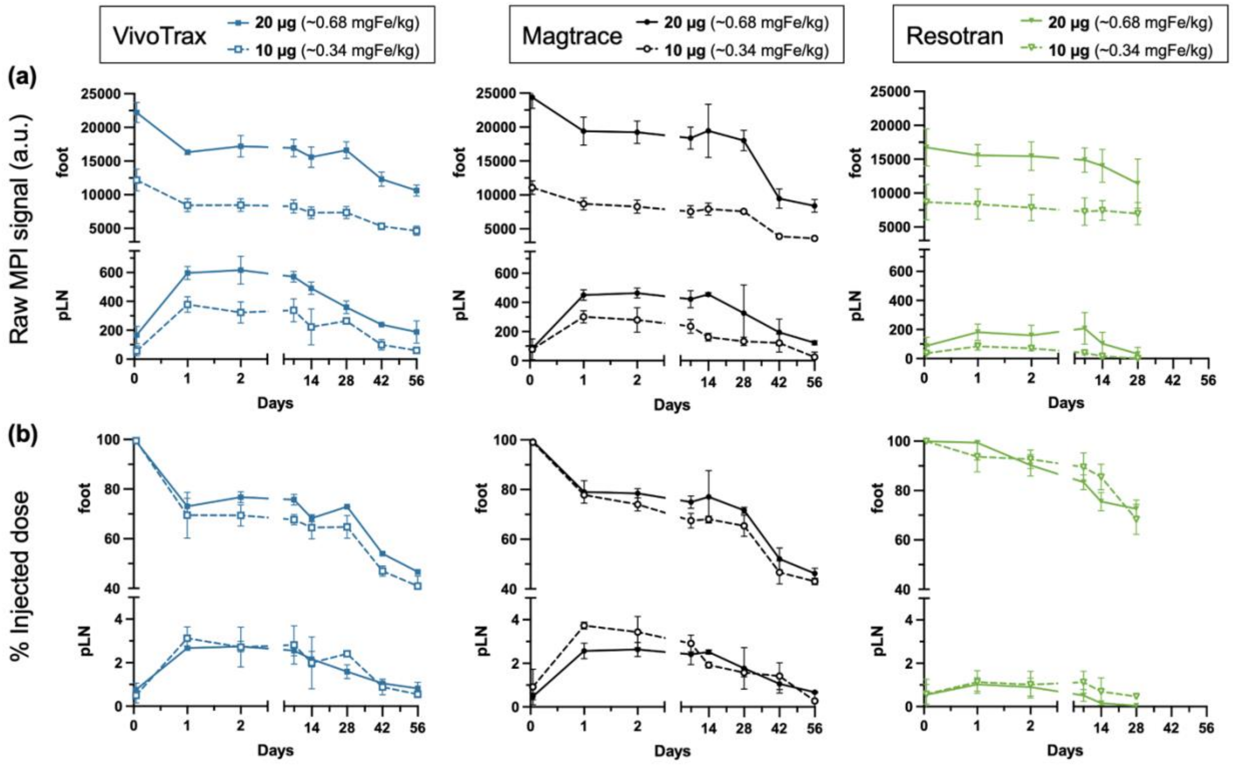

Figure S2. Dose study of VivoTrax, Magtrace, and Resotran in healthy lymphatics. Quantification of MPI signal at popliteal lymph nodes (pLNs) and injection site (footpads) after receiving 20 µg Fe (~0.68 mg Fe/kg, solid lines) compared with 10 µg Fe (~0.34 mg Fe/kg, dotted lines). Data expressed as **(a)** total (sum) MPI signal (arbitrary units, a.u.) and **(b)** percent injected dose (%ID). No significant differences in %ID were observed between the 10 µg Fe and 20 µg Fe dose levels for any tracer at either the injection site or pLN.

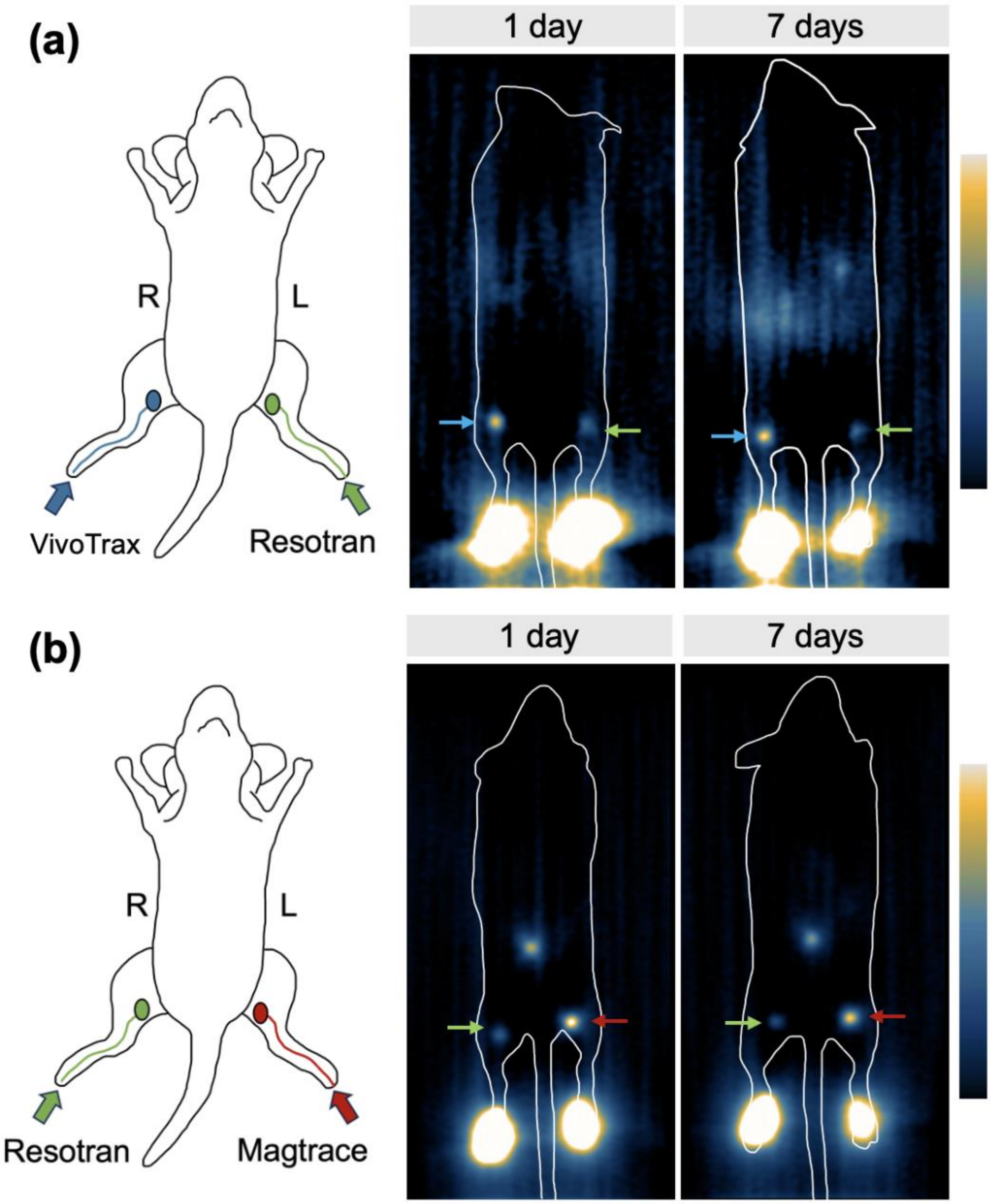

Figure S3. Direct comparison of *in vivo* pharmacokinetics of Resotran with VivoTrax and Magtrace by injection of 20 µg Fe into each footpad of the same mouse. (a) One C57 mouse received VivoTrax (right) and Resotran (left). **(b)** Another C57 mouse received Resotran (right) and Magtrace (left). Images were acquired 1 and 7 days after SPIO administration. Results consistently show relatively limited kinetics of Resotran to draining lymph nodes, compared with VivoTrax and Magtrace. The MPI signal in the abdomen reflects downstream lymphatic drainage of the bilaterally injected SPIOs, which first drain to the left and right popliteal lymph nodes and then continue to downstream left and right iliac lymph nodes.

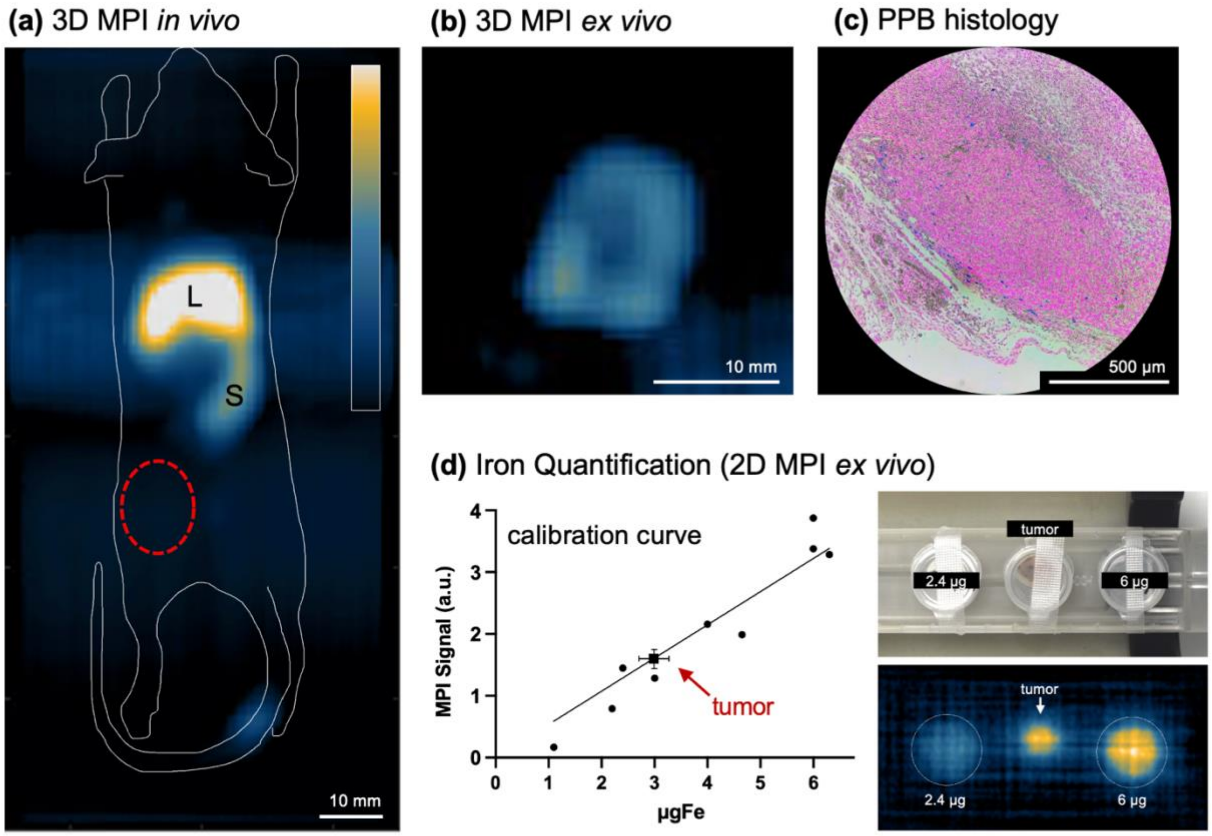

Figure S4. FerroTrace as an intravenous agent for *in vivo* labeling of tumor-associated macrophages (TAMs). Preliminary results shown from a balb/c mouse bearing a 4T1 breast tumor, imaged 1 day after intravenous infusion of 1.2 mg FerroTrace in 100 µL saline (n = 1). **(a)** Whole-body 3D MPI maximum intensity projection demonstrates predominant uptake of FerroTrace in the liver and spleen. The red circle indicates the expected tumor location *in vivo*. Image is displayed from 0–75% of the maximum signal. L = liver, S = spleen. **(b)** MPI of excised tumor shows signal localized primarily to the tumor periphery. Relative to panel (a), the image is magnified 2x and displayed using 100x lower signal range. 3D images are displayed using a model-based reconstruction algorithm (see methods). **(c)** Perls' Prussian blue (PPB) staining confirms the presence of iron deposits within the tumor tissue. **(d)** MPI signal quantification using 9 calibration samples (1.1 – 6.3 µg Fe) indicates 3.0 $\pm$ 0.3 µg Fe was present in the excised tumor, corresponding to ~0.25 %ID. **(e)** Linear calibration curve relating FerroTrace mass to MPI signal, generated from calibration samples (circles) and used to estimate iron mass in the tumor (square). Quantification was performed on 2D images reconstructed by the X-Space formulation.

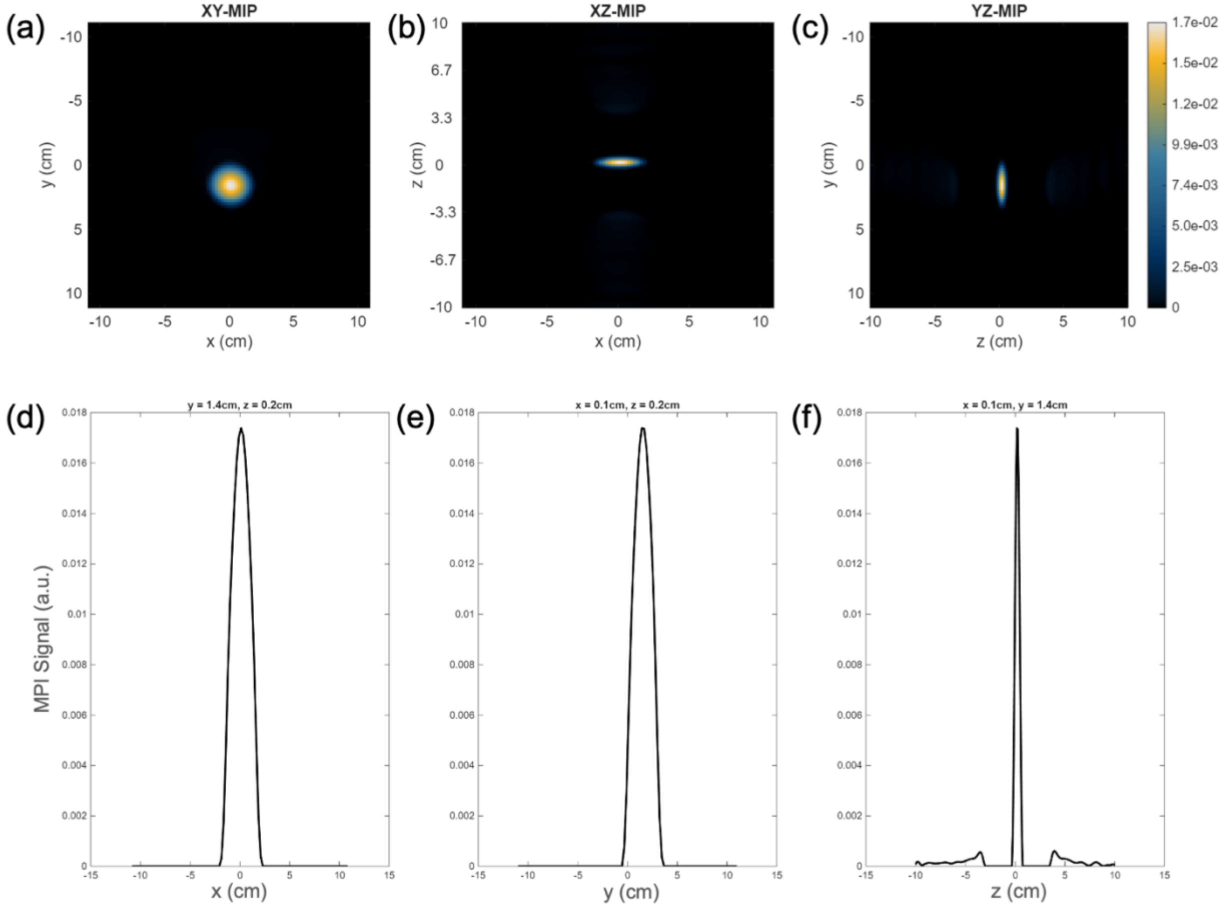

Figure S5. Clinical-scale imaging of Magtrace. A Magtrace sample containing 7 mg Fe in 0.25 mL was positioned at x = 0.1 cm, y = 1.4 cm, z = 0.2 cm and imaged on the clinical-scale MPI system. MPI scans are displayed as maximum intensity projections (MIP) in the **(a)** axial plane (XY), **(b)** coronal plane (XZ) and **(c)** sagittal plane (YZ). Line profiles show the MPI signal as a function of distance in the **(d)** x-direction (Left-Right), **(e)** y-direction (Anterior-Posterior), and **(f)** z-direction (Superior-Inferior).
